# Secretome Analysis of Mesenchymal Stem Cell Factors Fostering Oligodendroglial Differentiation of Neural Stem Cells In Vivo

**DOI:** 10.3390/ijms21124350

**Published:** 2020-06-18

**Authors:** Iria Samper Agrelo, Jessica Schira-Heinen, Felix Beyer, Janos Groh, Christine Bütermann, Veronica Estrada, Gereon Poschmann, Ana Bribian, Janusz J. Jadasz, Laura Lopez-Mascaraque, David Kremer, Rudolf Martini, Hans Werner Müller, Hans Peter Hartung, James Adjaye, Kai Stühler, Patrick Küry

**Affiliations:** 1 Department of Neurology, Medical Faculty, Heinrich-Heine-University, D-40225 Düsseldorf, Germany; iria.samperagrelo@med.uni-duesseldorf.de (I.S.A.); j.schira@uni-duesseldorf.de (J.S.-H.); fel.beyer@fau.de (F.B.); christine.buetermann@med.uni-duesseldorf.de (C.B.); veronica.estrada@uni-duesseldorf.de (V.E.); janusz.jadasz@uni-duesseldorf.de (J.J.J.); david.kremer@med.uni-duesseldorf.de (D.K.); hanswerner.mueller@uni-duesseldorf.de (H.W.M.); hans-peter.hartung@uni-duesseldorf.de (H.P.H.); 2 Institute for Biochemistry, University of Erlangen-Nürnberg, 91054 Erlangen, Germany; 3 Department of Neurology, Section of Developmental Neurobiology, University Hospital Würzburg, D-97080 Würzburg, Germany; groh_J@ukw.de (J.G.); rudolf.martini@mail.uni-wuerzburg.de (R.M.); 4 Institute for Molecular Medicine, Proteome Research, Medical Faculty, Heinrich-Heine-University, D-40225 Düsseldorf, Germany; gereon.poschmann@uni-duesseldorf.de (G.P.); kai.stuehler@uni-duesseldorf.de (K.S.); 5 Departamento de Neurobiología Molecular, Celular y del Desarrollo, Instituto Cajal-CSIC, 28002 Madrid, Spain; abribian@gmail.es (A.B.); mascaraque@cajal.csic.es (L.L.-M.); 6 Institute for Stem Cell Research and Regenerative Medicine, Medical Faculty, Heinrich-Heine-University, D-40225 Düsseldorf, Germany; james.adjaye@med.uni-duesseldorf.de; 7 Molecular Proteomics Laboratory (MPL), Biologisch-Medizinisches Forschungszentrum (BMFZ), Heinrich-Heine-University, D-40225 Düsseldorf, Germany

**Keywords:** neural stem cells, mesenchymal stem cells, transplantation, oligodendroglia, glial fate modulation, myelin, spinal cord, secretome, TIMP-1

## Abstract

Mesenchymal stem cell (MSC)-secreted factors have been shown to significantly promote oligodendrogenesis from cultured primary adult neural stem cells (aNSCs) and oligodendroglial precursor cells (OPCs). Revealing underlying mechanisms of how aNSCs can be fostered to differentiate into a specific cell lineage could provide important insights for the establishment of novel neuroregenerative treatment approaches aiming at myelin repair. However, the nature of MSC-derived differentiation and maturation factors acting on the oligodendroglial lineage has not been identified thus far. In addition to missing information on active ingredients, the degree to which MSC-dependent lineage instruction is functional in vivo also remains to be established. We here demonstrate that MSC-derived factors can indeed stimulate oligodendrogenesis and myelin sheath generation of aNSCs transplanted into different rodent central nervous system (CNS) regions, and furthermore, we provide insights into the underlying mechanism on the basis of a comparative mass spectrometry secretome analysis. We identified a number of secreted proteins known to act on oligodendroglia lineage differentiation. Among them, the tissue inhibitor of metalloproteinase type 1 (TIMP-1) was revealed to be an active component of the MSC-conditioned medium, thus validating our chosen secretome approach.

## 1. Introduction

In demyelinating diseases of the central nervous system (CNS), such as multiple sclerosis, myelin repair activities can be observed on the basis of recruitment, activation, and differentiation of resident precursor and stem cells [1]. Besides oligodendroglial precursor cells (OPCs), multipotent adult neural stem cells (aNSCs) located in the subventricular zone (SVZ) or the dentate gyrus of the hippocampus serve as an additional source for the generation of myelinating oligodendrocytes [2,3,4,5,6,7]. Yet, their regeneration capacity and overall degree of remyelination remains limited, which is likely a result of multiple extrinsic and intrinsic inhibitory factors [8,9,10,11,12,13,14]. Moreover, immune reactions and damaged tissues create an overall hostile environment, which further limits regeneration activities [15]. Insights into the underlying mechanisms on how aNSCs can be stimulated to differentiate into a specific cell lineage could therefore pave the way for novel neuroregenerative treatment approaches, which are still representing a clinically unmet need.

Mesenchymal stem cells (MSCs) are known for their immune-modulatory capacity and can also mediate a number of regenerative responses [16]. Regarding the restricted repair potential of the adult CNS, rodent and human MSCs as well as their secreted factors have repeatedly been shown to promote oligodendrogenesis from aNSCs and OPCs [16,17,18,19,20]. The corresponding factors are likely secreted or shedded, and their identification might therefore provide important information on how existing limitations regarding successful myelin repair might be overcome. However, the nature of MSC-derived differentiation and maturation factors acting on the oligodendroglial lineage have not been disclosed thus far [21] and might therefore to be deciphered by sophisticated strategies such as mass spectrometry-based secretome analysis [22]. Although MSC secretome and proteome data already exist, most of them deal with their anti-inflammatory properties [23,24,25]. In addition to the missing information on active ingredients, it also remains to be demonstrated to what degree MSC-dependent lineage instruction is functional in vivo.

In the present study, we demonstrate that mesenchymal stem cell factors can indeed stimulate aNSC-derived oligodendrogenesis in different CNS regions and we furthermore provide insights into the underlying mechanisms as we conducted a comparative secretome and proteome analyses. We identified several secreted proteins, many of which have unknown roles in the glial context. However, the analysis also revealed a group of proteins that were previously shown to act on oligodendroglia.

## 2. Results

### 2.1. Survival of MSC-CM-Stimulated aNSC In Vivo

Mesenchymal stem cell secreted factors were shown to stimulate aNSCs towards an oligodendrogenic differentiation by yet unknown mechanisms [17,19,20]. To investigate the potential of rat MSC-conditioned medium (MSC-CM) stimulated aNSCs to generate oligodendroglial cells, we performed in vivo cell transplantations into different CNS tissues. Before transplantation experiments were initiated, we pre-treated green fluorescent protein (GFP)-expressing aNSCs were with either control α-minimum essential medium containing 10% fetal calf serum (α-MEM/10% FCS) or MSC-CM, and tested for the expression of oligodendroglial markers after 1, 3, and 7 days in vitro. These analyses revealed a significant increase (up to 15%) in the number of GFP-positive cells upon incubation in both media over time (Figure S1). In addition, a significantly increased number of cells expressing oligodendroglial markers was observed after MSC-CM stimulation, whereas astrocytic marker expression was decreased in MSC-CM-treated aNSCs compared to control medium-treated cells (Figure S1), thus reproducing MSC-CM-dependent effects previously shown in non-transfected cells [20].

**Figure 1:**
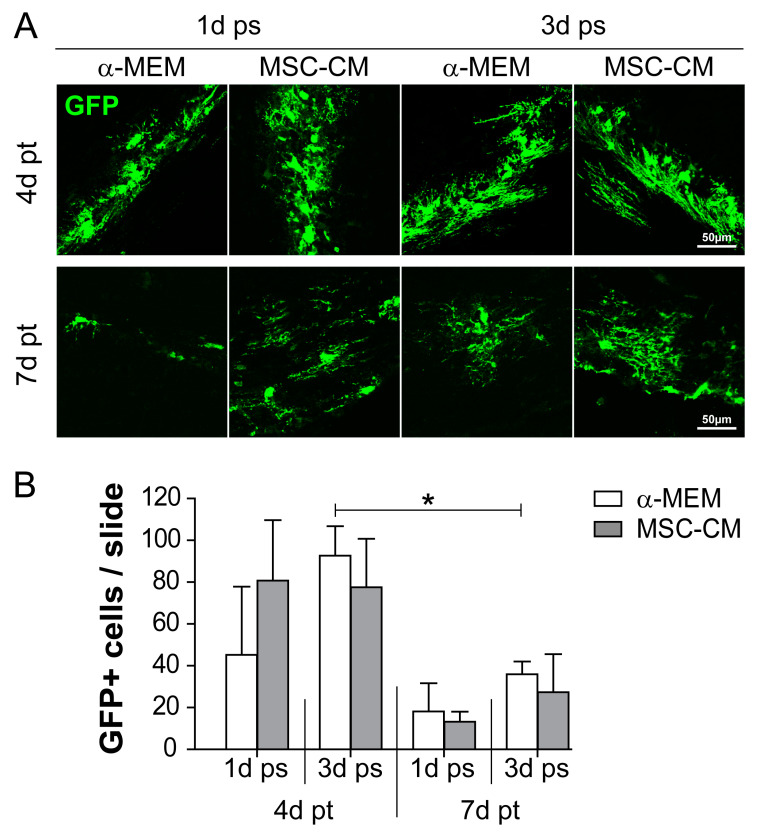
Survival rate of green fluorescent protein (GFP)-expressing cells after transplantation into the mouse brain. (**A**) Immunohistochemical staining was used to identify transplanted GFP-positive adult neural stem cells (aNSCs) (green) at 4 and 7 days post-transplantation (pt) into the mouse brain. aNSCs were pre-stimulated (ps) for either 1 or 3 days (1d ps and 3d ps, respectively) with α-minimum essential medium (α-MEM) or mesenchymal stem cell (MSC)-conditioned medium (MSC-CM). Representative pictures were taken from white (corpus callosum) / grey (cortex) matter implantation sites. (**B**) Quantitative evaluation of the total number of GFP-positive cells per slide including both grey and white matter. Statistical significance was calculated using a two-way ANOVA with Bonferroni posttest: * *p* ≤ 0.05. Data were generated on the basis of the following animal numbers (*n*): 1d ps and 4d pt, α-MEM and MSC-CM *n* = 4; 3d ps and 4d pt, α-MEM *n* = 5 and MSC-CM *n* = 4; 1d ps and 7d pt, α-MEM and MSC-CM *n* = 3; 3d ps and 7d pt, α-MEM and MSC-CM *n* = 4.

To investigate to what degree MSC-CM-stimulated aNSCs differentiate and integrate within different brain environments, we performed transplantations into either grey (GM) or white matter (WM) regions of young adult mouse brains as previously described [26]. Moreover, we tested different MSC-CM stimulation periods prior to transplantation. We first monitored to what extent GFP-expressing cells survive after transplantation and to what level stimulation with either control α-MEM or with MSC-CM affects cell survival in vivo. Transplantation into the grey (cortex) and white (corpus callosum) matter of the mouse brain revealed that transplanted aNSCs survived up to 7 days post-grafting, independent of their pre-stimulation (duration and condition). In addition, more GFP-positive cells were found after 4 days compared to 7 days post-transplantation (pt). A 3-day MSC-CM pre-stimulation period appeared to confer a slight survival benefit compared to the 1-day pre-treatment (Figure 1). Additionally, no significant differences were observed when cell survival between grey and white matter sites were compared (Figure S2). Our analysis also revealed that the survival rate of transplanted aNSCs decreased as nearly no GFP-positive cells were found at 14 days post-transplantation (data not shown).

### 2.2. MSC-Derived Factors Promoted the Oligodendroglial Differentiation Process In Vivo

**Figure 2:**
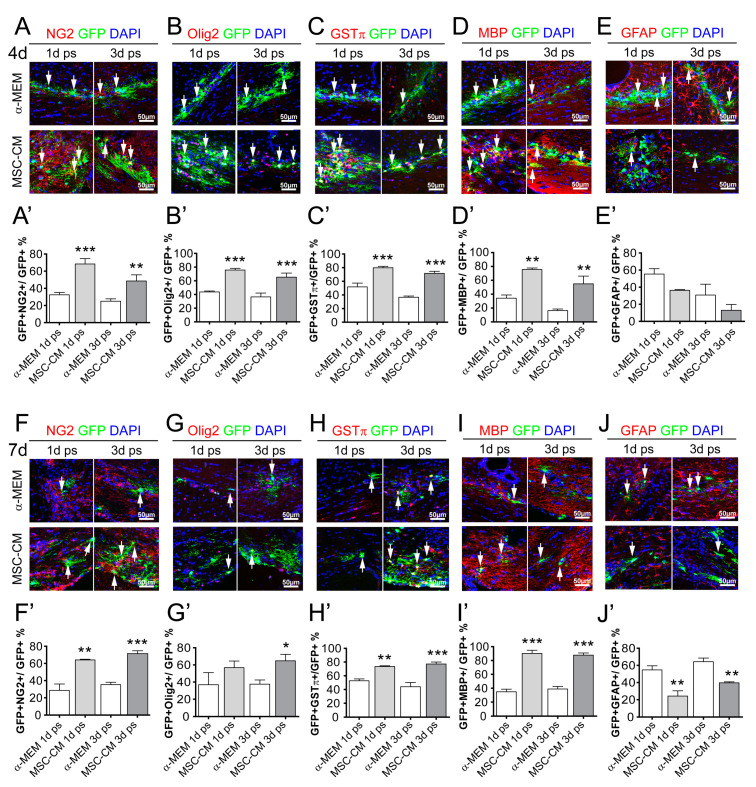
MSC-CM pre-stimulated aNSCs differentiated into mature myelin basic protein (MBP)-expressing oligodendrocytes after transplantation into the mouse brain. (**A**–**E**) Representative images of control (α-MEM) and MSC-CM pre-stimulated and transplanted GFP-positive aNSCs expressing neural/glial antigen 2 (NG2) (**A**), oligodendrocyte transcription factor 2 (Olig2) (**B**), glutathione-S-transferase-π (GSTπ) (**C**), MBP (**D**), or glial fibrillary acidic protein (GFAP) (**E**) 4 days after transplantation into the brain. Quantification of transplanted cells expressing NG2 (**A’**), Olig2 (**B’**), GSTπ (**C’**), and MBP (**D’**) revealed a significantly increased number of cells expressing oligodendroglial markers after 4 days, whereas the degree of GFAP-positive cells (**E’**) was significantly decreased. The same effects were observed at 7 days post-transplantation (**F**–**J**) with the corresponding quantifications shown for NG2 (**F’**), Olig2 (**G’**), GSTπ (**H’**), MBP (**I’**), and GFAP (**J’**). For statistical analysis, a two-way ANOVA with Bonferroni posttest was used: * *p* ≤ 0.05, ** *p* ≤ 0.01, *** *p* ≤ 0.001, MSC-CM compared to the respective α-MEM control (1- or 3-day pre-stimulation). Arrows point to GFP-positive cells (green) expressing the respective markers (red). Blue nuclei represent 4´,6-diamidin-2-phenylindol (DAPI) staining. Animal numbers (*n*): 3–5.

We next investigated the differentiation capacity of transplanted aNSCs after 4 and 7 days in the mouse brain. To this end, we examined expression of markers for OPCs, oligodendrocytes, and astrocytes in GFP-positive cells. MSC-CM pre-treatment led to an increase in neural/glial antigen 2 (NG2)-positive cells after 4 and 7 days compared to aNSCs that were pre-stimulated with α-MEM, (Figure 2A,A’,F,F’). Importantly, the degree of NG2 induction was similar between cells that were only stimulated for 1 day and for cells stimulated for 3 days (compare grey bars in Figure 2A’,F’). Likewise similar induction rates were observed for further markers along the oligodendroglial lineage such as oligodendrocyte transcription factor 2 (Olig2) (Figure 2B,B’,G,G’), glutathione-S-transferase-π (GSTπ) (Figure 2C,C’,H,H’), and myelin basic protein (MBP) (Figure 2D,D’,I,I’) at both investigated time points. Similar to the observed differentiation kinetics of cultured aNSCs [20], accumulation of oligodendroglial markers was accompanied by a MSC-CM dependent decrease of glial fibrillary acidic protein (GFAP)-positive cells (Figure 2E,E’,J,J’). When the differentiation capacity of transplanted cells in grey and white matter was compared, no significant difference was observed (Figure S3). Our data therefore indicate that a 1-day long pre-stimulation with MSC-derived factors is sufficient to boost the oligodendroglial differentiation process of aNSCs after transplantation in vivo. However, for practical reasons, we continued using a 3-day pre-stimulation period for aNSCs to be implanted into spinal cords.

### 2.3. Extended Survival Time of MSC-CM Pre-Stimulated aNSCs Transplanted into the Adult Rat Spinal Cord

To evaluate the survival rate of MSC-CM pre-stimulated aNSCs and their differentiation potential after transplantation into other CNS tissues, we transplanted MSC-CM stimulated aNSCs into the grey or white matter of adult rat spinal cords. In this regard, 3-day pre-stimulated aNSCs were analyzed at 14 and 28 days after transplantation in order to monitor tissue integration, cell fate acquisition, maturation stages, and myelination capacities.

**Figure 3:**
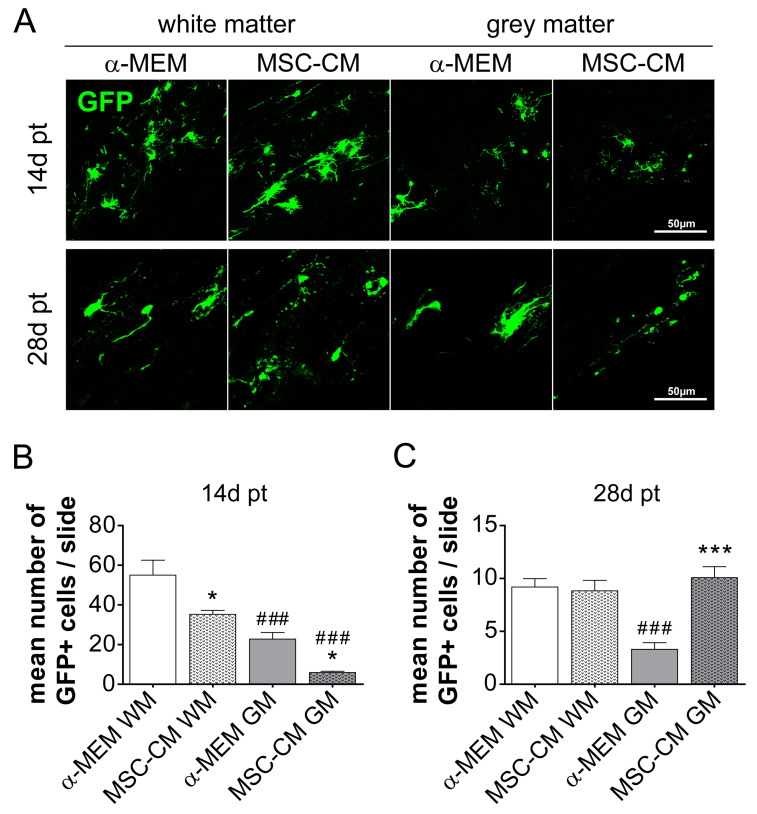
Survival rate of rat aNSCs after transplantation into the rat spinal cord. (**A**) Representative images of GFP-positive transplanted cells 14 and 28 days post-transplantation (pt) either in white or grey matter of the rat spinal cord. Corresponding quantifications of GFP-positive cells per slide (a mean of three sections per slide) and animal in white and grey matter at 14 days (**B**) and 28 days (**C**). Statistical significance was calculated using a two-way ANOVA with Bonferroni posttest: * *p* ≤ 0.05, *** *p* ≤ 0.001 (for comparison between control α-MEM to MSC-CM) and ^###^ *p* ≤ 0.001 (for comparison between white and grey matter and respective transplantation paradigm). Animal numbers (*n*): 14 days pt α-MEM *n* = 4 and MSC-CM *n* = 5, 28 days pt α-MEM *n* = 6 and MSC-CM *n* = 4.

To evaluate the survival rate of transplanted cells, we counted the number of GFP-positive cells per slide and animal at both time points. Quantification of transplanted cells revealed that after 14 days the white matter environment exerted a general survival benefit on implanted cells. Furthermore, in both grey as well as white matter regions, fewer MSC-CM-stimulated cells compared to control (α-MEM)-stimulated cells were observed (Figure 3A,B). However, at the later time point (28 days), we observed no treatment difference in the white matter, while in the grey matter, MSC-CM pre-stimulated cells dominated over control (α-MEM)-treated cells.

**Figure 4:**
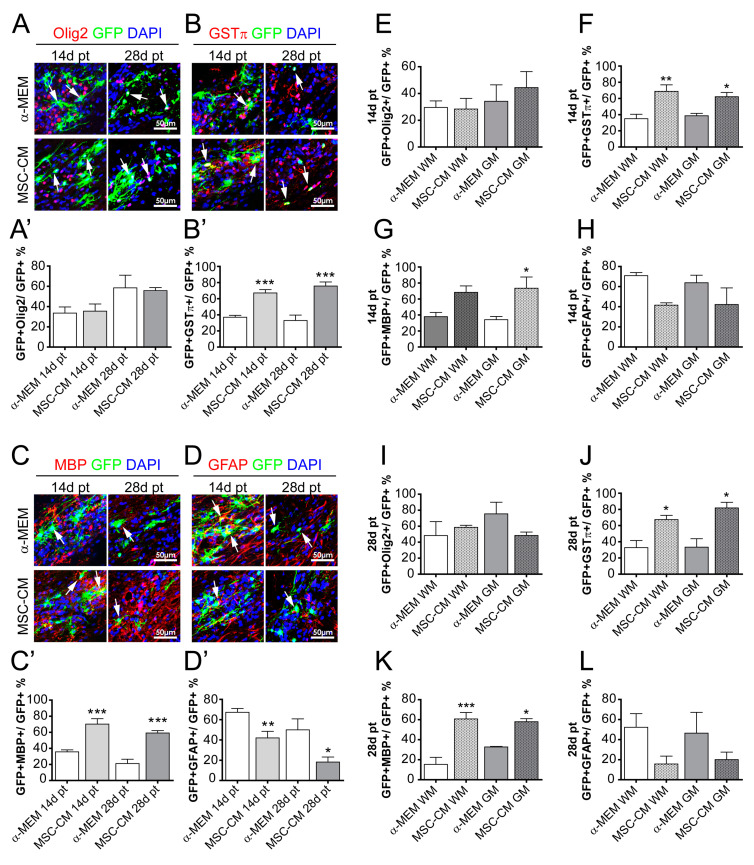
Accumulation of oligodendroglial characteristics in spinal cord-transplanted aNSCs. Representative pictures of GFP-positive cells expressing Olig2 (**A**), GSTπ (**B**), MBP (**C**), and GFAP (**D**) at 14 and 28 days post-transplantation and following α-MEM or MSC-CM pre-treatment. The corresponding quantifications (percentage of expressing cells over total number of GFP-expressing cells) are depicted in (**A’**) for GFP/Olig2- in (**B’**) for GFP/GSTπ-, in (**C’**) for GFP/MBP-, and in (**D’**) for GFP/GFAP-positive cells. (**E**–**L**) Quantification and distribution of transplanted GFP-positive cells pre-treated with α-MEM and MSC-CM within white matter (WM) and grey (GM) at 14 and 28 days post-transplantation. Statistical significance was calculated using a two-way ANOVA with Bonferroni posttest: * *p* ≤ 0.05, ** *p* ≤ 0.01, *** *p* ≤ 0.001 (for comparison between control α-MEM to MSC-CM). Arrows point to GFP-positive cells (green) expressing the respective marker proteins (red). Blue nuclei represent DAPI staining. Animal numbers (*n*): 4–6.

Investigating the differentiation capacity and maturation of spinal cord-transplanted cells confirmed an enhanced oligodendroglial cell fate upon MSC-CM pre-stimulation at 14 and 28 days post-transplantation. Although no impact of MSC-CM pre-stimulation on the degree of Olig2-positive cells was observed (Figure 4A,A’), the degree of GSTπ-positive and MBP-positive cells was strongly boosted upon MSC-CM pre-treatment at both time points (Figure 4B,B’,C,C’). Similar to the brain-transplanted cells, this increase in oligodendrogenesis was accompanied by an MSC-CM-dependent decrease in GFAP positivity among GFP-positive cells (Figure 4D,D’). This pro-oligodendroglial behavior did not substantially differ between WM- and GM-transplanted cells (Figure 4E–L). In addition, a potential neuronal differentiation capacity was tested by means of neuronal nuclei antigen (NeuN) and neurofilament (NF) immunohistochemical staining. No GFP/NeuN or GFP/NF-positive cells were found under both conditions (α-MEM or MSC-CM pre-stimulation) and at both time points (Figure S4). These data therefore confirmed a strong pro-oligodendroglial effect exerted by MSC-derived factors and at the same time revealed increased numbers of surviving and integrated cells in the spinal cord.

### 2.4. MSC-CM Pre-Stimulation Enabled aNSCs to Myelinate Axons In Vivo

**Figure 5:**
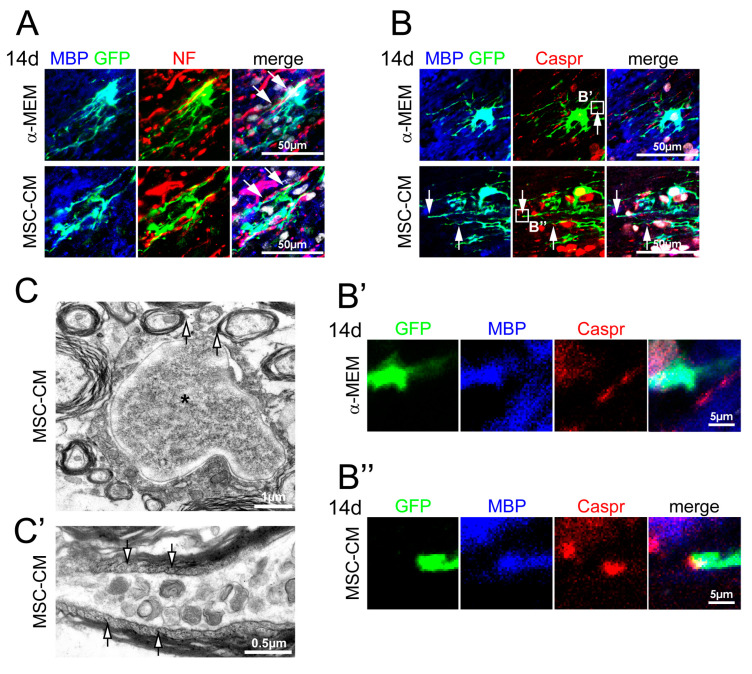
MSC-CM pre-treated aNSCs were able to generate myelinated segments. (**A**) Representative pictures of MBP-/GFP-/neurofilament-stained spinal cord sections at 14 days post-transplantation. For both α-MEM and MSC-CM pre-treated and transplanted cells, we observed an alignment of MBP-positive segments with neurofilament (NF)-positive axons (arrows). Nuclei are depicted in white. (**B**) MBP-positive processes of MSC-CM pre-stimulated cells often ended in Caspr-positive paranodes (arrows). Nuclei are depicted in white. Higher magnifications are depicted in (**B’**) for α-MEM and in (**B’’**) for MSC-CM pre-stimulated aNSCs. (**C**) Representative electron micrograph of a GFP-positive cells (asterisk: nucleus) 14 days post-transplantation. Chromogenic GFP-immunoreactivity (dark precipitate) identified myelinating processes (arrows). (**C’**) GFP expression was also detectable in the cytoplasm of paranodal loops (arrows).

In order to analyze the functionality of MBP-expressing cells and to identify myelinated segments, we performed co-staining for neurofilament and contactin-associated protein (Caspr), a marker for paranodal axonal regions of nodes of Ranvier, after 14 days of cell transplantation into rat spinal cords as previously established for other stem cell transplantation approaches [27,28]. Immunohistochemical staining revealed that processes of transplanted GFP/MBP-double positive cells pre-stimulated with α-MEM or MSC-CM often aligned with neurofilament-positive axons (Figure 5A). Triple staining for GFP, MBP, and Caspr revealed that processes of GFP/MBP-positive cells abutted at Caspr-positive paranodal regions (Figure 5B,B’’). Importantly, such myelinated segments and nodal structures were only observed at MSC-CM pre-treated NSCs, thus providing strong evidence that mesenchymal factors contribute to the generation of myelinated segments in vivo. Moreover, ultrastructural analysis of transplanted MSC-CM pre-treated cells by means of immunoelectron microscopy (as established in [26]; transplanted cells were identified by chromogenic anti-GFP immunohistochemistry resulting in dark precipitates), demonstrated that these cells established myelin sheaths around axons (Figure 5C) and also revealed GFP-immunoreactive signals in paranodal loops (Figure 5C’).

### 2.5. Characterization of Adult Rat Mesenchymal Stem Cell Secretome

**Figure 6:**
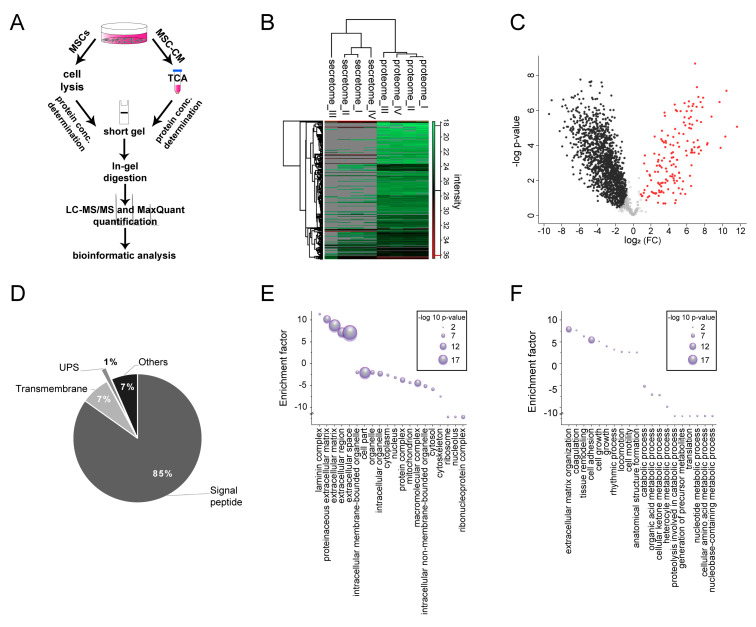
Characterization of the mesenchymal stem cell secretome. (**A**) Workflow for preparation of MSC-derived proteomes and secretomes for quantitative LC–MS/MS analysis. (**B**) Heatmap of proteins quantified in the MSC secretome and proteome revealed a high similarity between the proteome samples that clearly differed from the secretomes (grey = proteins were not identified). (**C**) Volcano plot showing proteins found to be enriched in the MSC-derived secretome (152 secretome-enriched proteins, shown as red dots on right side) and the proteome (left side, black dots). (**D**) Out of the 152 proteins found to be enriched in the secretome, 85% of them were predicted to have a signal peptide, 7% were transmembrane proteins, and 1% were unconventionally secreted (UPS). (**E**) Gene Ontology (GO) enrichment analysis of cellular component revealed that these proteins were associated with extracellular space and matrix localization. (**F**) GO enrichment analysis of biological process revealed an association with extracellular matrix organization and cell adhesion (5% false discovery rate (FDR)).

Despite several approaches to identify active components, the underlying mechanism and acting factors of MSC-mediated pro-oligodendroglial effects is still unknown. In order to shed light onto the composition of active proteins in the MSC secretome and to identify MSC secreted pro-oligodendroglial proteins, we performed a mass spectrometry-based secretome analysis (Figure 6). For mass spectrometry analysis, MSCs and corresponding conditioned media were harvested after incubation with serum-free N2-medium for 48 h. Proteins of four independent replicates were processed and analyzed with liquid chromatography–mass spectrometry (LC–MS/MS; Figure 6A). Secretomes were compared to corresponding proteomes in order to identify bona fide secreted proteins and subtract potential contaminations from dying cells due to higher abundances in the secretome (Figure 6B; Table S1) (according to [29]). Thus, from the total number of proteins (691 proteins) identified in MSC-CM, we detected 152 proteins as bona fide secreted due to the comparison with the cellular proteome (Figure 6C; Table S1). From all secreted proteins, 85% of the proteins were predicted to have a signal peptide assigning them to the classical secretory pathway, 7% exhibited a transmembrane domain, and 1% were presumably unconventionally secreted proteins (Figure 6D). Categorical enrichment analysis revealed that bona fide secreted proteins were associated with extracellular components such as the laminin complex (enrichment factor (EF) 11.4, *p*-value 6.50 × 10^−4^), the proteinaceous extracellular matrix (EF 10.3, *p*-value 5.6 × 10^−18^), the extracellular matrix (EF 8.9, *p*-value 3.3 × 10^−30^), and extracellular space (EF 7.2, *p*-value 8.8 × 10^−25^) (Figure 6E), whereas categories related to intracellular compartments such as the nucleus (EF -3.0, *p*-value 4.7 × 10^−6^), the macromolecular complex (EF −4.3, *p*-value 1.2 × 10^−16^), and the ribonucleoprotein complex (EF −12.0, *p*-value 7.8 × 10^−8^) were under-represented (Figure 6E). Furthermore, MSC-secreted proteins were associated with biological processes related to extracellular compartments such as the extracellular matrix organization (EF 7.9, *p*-value 1.0 × 10^−15^), cell adhesion (EF 5.5, *p*-value 5.9 × 10^−18^), and locomotion (EF 2.7, *p*-value 7.4 × 10^−4^) (Figure 6F), whereas proteins related to intracellular processes such catabolic processes (EF −5.2, *p*-value 1.4 × 10^−6^) and translation (EF −12, *p*-value 2.6 × 10^−3^) were under-represented. We further analyzed these 152 proteins regarding a pro-oligodendroglial fate choice (Table 1). According to the already described functions, several proteins identified in the MSC secretome can either directly or indirectly be implicated in oligodendroglial differentiation or myelination. This includes, for example, extracellular matrix proteins such as laminin subunit beta-2 (Lamb2), tissue inhibitor of metalloproteinase inhibitor type 1 (TIMP-1), dystroglycan (Dag1), aggrecan (Acan), or bone morphogenetic protein (BMP)-associated proteins such as chordin (Chrd) and metalloendopeptidase (BMP-1). Moreover, growth factors acting as possible negative regulators such as connective tissue growth factor (CTGF) and insulin-like growth factor-binding proteins (IGFBP).

### 2.6. Neutralization of TIMP-1 Diminished the Pro-Oligodendroglial Effect of MSC-CM 

**Figure 7:**
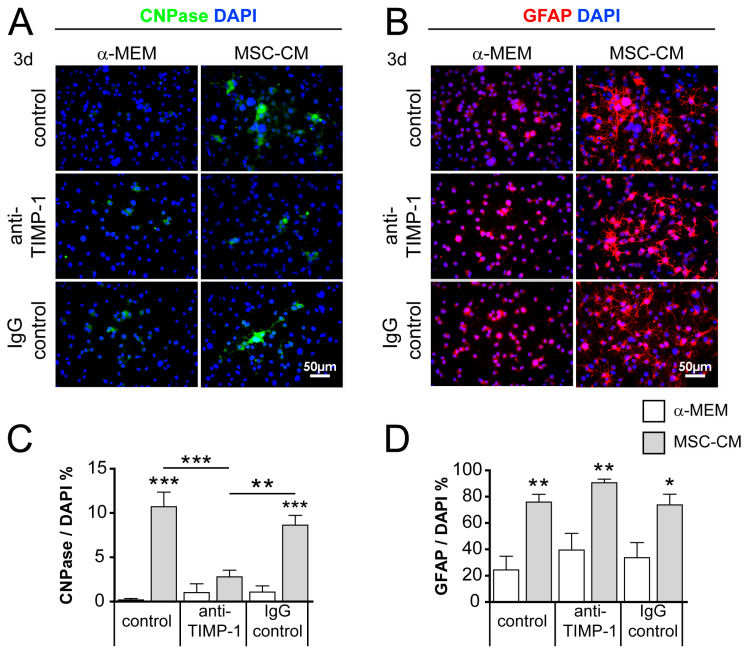
Blocking the tissue inhibitor of metalloproteinase type 1 (TIMP-1) in MSC-CM reduced its pro-oligodendroglial activity on cultured aNSCs. Representative immunocytochemical staining of 2′,3′-cyclic-nucleotide 3′-phosphodiesterase (CNPase)- (**A**) and GFAP-positive (**B**) cells treated either with control (no antibody), anti-TIMP-1, or immunoglobulin G (IgG) control pre-incubated with α-MEM or MSC-CM after 3 days in culture. (**C**) Quantification of CNPase- and GFAP-positive (**D**) cell numbers. Number of experiments *n* = 4. Statistical significance was calculated using a two-way ANOVA with Bonferroni posttest: * *p* ≤ 0.05, ** *p* ≤ 0.01, *** *p* ≤ 0.001.

Proteins found to be enriched in the MSC secretome included tissue inhibitor of metalloproteinase type 1 (TIMP-1), which represents a promising candidate protein with known pro-oligodendroglial properties [42,43,50]. In order to validate our protein identification approach, we performed specific TIMP-1 antibody blocking assays on cultured aNSCs. To this end, we applied the antibody to α-MEM and MSC-CM for 1 h prior to application of aNSCs. For further control, no antibody (normal control) and immunoglobulin G (IgG) isotype control antibody was applied. After 3 days of culture, we determined the degree of 2′,3′-cyclic-nucleotide 3′-phosphodiesterase (CNPase)-positivity among aNSCs. Neutralization of TIMP-1 in MSC-CM resulted in a significantly reduced number of CNPase-positive cells compared to untreated (control) MSC-CM, as well as to IgG antibody control-treated MSC-CM (Figure 7). After TIMP-1 neutralization, we further demonstrated that the number of GFAP-positive cells tended to increase compared to normal control and IgG antibody controls (Figure 7C). To exclude intrinsic TIMP-1 antibody effects, we also used a series of unconditioned α-MEM, which revealed no TIMP-1 or IgG control antibody effects on CNPase expression or GFAP-positive cells. 

## 3. Discussion

In demyelinating diseases, neural stem cells can be actively recruited for myelin repair [1,3,4,5,6,7]. However, despite of their ability to differentiate into oligodendrocytes, the extent of regeneration and remyelination in the adult CNS remains limited [11,12,13]. Thus, insights into the underlying mechanisms on how aNSCs can be fostered to differentiate into a specific cell lineage could help in establishing neuroregenerative treatment approaches, which are still a clinically unmet need. Forthcoming strategies could include the biological or pharmacological manipulation of endogenous aNSCs or, under specific circumstances, also the transplantation of aNSCs previously primed to acquire oligodendroglial cell fates. This second approach would nevertheless be restricted to well-described white matter lesions. However, in an experimental set-up, site-directed cell transplantation might indeed provide valuable information on how stem cells can be modulated and placed specifically in order to regenerate oligodendrocytes and white matter. Previous studies demonstrated that rodent and human MSC-secreted factors promote oligodendroglial differentiation of aNSCs in vitro [17,19,20,51]. However, the degree to which MSC-dependent lineage instruction is functional in vivo remains to be demonstrated. Moreover, the nature of MSC-derived differentiation factors regarding oligodendroglial lineage differentiation remain elusive thus far [21].

### 3.1. Enhanced Oligodendroglial Differentiation of MSC-CM-Stimulated aNSCs In Vivo

Our present study revealed that aNSC transplanted into different CNS tissues are able to survive at least up to 28 days post-grafting, independent of their pre-treatment. As expected, cell survival was diminished over time, which could depend on the immune response since the animals were not immune-suppressed as in other studies [52,53,54]. In comparison to the brain, the spinal cord environment appeared to provide a better survival rate. In this regard, it should be noted that this difference might also derive from rat cells being implanted into mouse brains, which could cause additional immune responses. Given that mouse neural stem cells have thus far not been shown to respond adequately to MSC-derived factors (own observations), we could not apply a direct comparison using a mouse-to-mouse transplantation scheme.

In the brain, grey and white matter can differently affect OPC differentiation [55], and we previously showed that white matter localization promotes differentiation towards myelinating oligodendrocytes at the expense of astrocyte generation when transplanted aNSC were depleted from the intrinsic oligodendrogenesis inhibitor p57kip2 [26]. However, in both studies, regional differences of the brain showed no impact on cell survival, which was confirmed by the here-described brain transplantation experiments. While transplantation of aNSCs into grey or white matter of the brain showed no difference in survival, most of the transplanted cells were found in white matter after spinal cord transplantation. It therefore appears that spinal cord white matter constitutes a preferred region for transplanted cells, which is in agreement with previous observations [56]. 

More importantly, our experiments clearly revealed that MSC-derived factors can indeed boost the generation of oligodendrocytes in vivo while preventing an astrocytic cell fate. Whereas such a directed stimulation depends on at least 3 days of pre-stimulation in cultured cells [20], our current results demonstrate that the pro-oligodendroglial effect in vivo can be equally well seen after a 1-day pre-treatment period. It thus appears that such an initial MSC-dependent stimulation of oligodendrogenesis is dominant and the corresponding impulse can be maintained in the CNS environment.

Besides the predominant expression of oligodendroglial markers, we only noticed myelin sheath generation in the spinal cord in response to pre-stimulation with mesenchymal-derived factors, indicating that not only quantitative but also qualitative effects are mediated. Myelin sheath generation was revealed by the presence of paranodal Caspr signals in close apposition to MBP-positive processes of MSC-CM pre-stimulated and transplanted cells [27,28]. Furthermore, representative examination of MSC-CM pre-stimulated cells by immunoelectron microscopy revealed myelinating processes ending in paranodal loops. This is furthermore supported by the observation that, at late time points, MSC-CM pre-treated aNSCs were more frequently observed in CNS tissues as these cells might have better survived due to successful tissue integration and axon ensheathment. Note that the generation of quantitative data on node formation and myelination is out of scope of this study and must be addressed in future investigations.

Although we detected more mature oligodendrocytes in response to MSC-CM, we found there was no difference in the number of Olig2-expressing cells, possibly reflecting the fact that Olig2-positivity, although differing in its subcellular distribution, is also featured by astrocytic cells [57].

The here-observed pro-oligodendroglial features within the almost healthy CNS (disregarding injection sites) suggest that similarly treated aNSCs might also be able to promote myelin repair upon injury and demyelination, also in light of the increased number of naked axons to be contacted. In addition, transplanted cells could also have an impact on endogenous progenitor or stem cells by direct cell–cell contact or by secretion of trophic factors, thus promoting endogenous remyelination capacity and functional regeneration. However, such experiments were out of the scope for the current analysis, but will be conducted in the future through applying toxin- as well as immune-mediated demyelination models.

### 3.2. MSCs Secreted a Number of Oligodendroglial Regulatory Proteins

Underlying pro-oligodendroglial mechanisms and proteins secreted by MSCs have not yet been deciphered in detail. Although some MSC secretome or proteome data already exist, most of them deal with anti-inflammatory, regenerative, and tumor-promoting properties of MSCs [23,24,25,58,59,60]. Therefore, we applied a quantitative secretome approach for the analysis of MSC-derived proteins with pro-oligodendroglial activity. From the initially identified 691 proteins, we determined 152 proteins (Table S1) as bona fide secreted due to the significant enrichment in MSC-CM. GO term enrichment analysis further confirmed that these proteins are mainly localized in the extracellular environment and predominantly involved in extracellular matrix (ECM) organization and cell adhesion. Moreover, in order to find pro-oligodendroglial factors, we further analyzed these 152 proteins regarding a pro-oligodendroglial fate choice (Table 1). On the basis of their previously shown impact on oligodendrogenesis, we identified first obvious promising candidates, among them ECM proteins such as Lamb2. Laminins were previously shown to be important for CNS myelination [61], and pericyte-dependent stimulation of aNSC-dependent oligodendrogenesis was recently found to depend on the related protein laminin subunit alpha 2 (Lama2) [62]. Laminins regulate CNS myelination by interacting with integrin receptors and by binding to the non-integrin ECM receptor dystroglycan [34], which was also enriched in the MSC secretome, possibly acting on OPC proliferation via its intracellular cleaved part [33]. Another bona fide secreted protein with a known pro-oligodendroglial function is chordin. As a BMP antagonist, it can promote oligodendrogenesis from SVZ neural stem cells [31], and we previously found chordin to be induced in response to the pro-oligodendroglial suppression of the p57kip2 inhibitor [63]. Furthermore, we detected syntenin-1, known to regulate OPC migration via interaction with NG2 [47]. While proteins such as dystroglycan and chordin promote proliferation and differentiation, prosaposin, also known as sulfated glycoprotein 1, promotes expression of myelin constituents and protects myelinating glial cells [45,46]. Protecting properties have also been assigned to the arrest-specific protein 6 (Gas6) [36], and its deficiency increases oligodendrocyte loss during cuprizone-induced demyelination [64]. Besides pro-oligodendroglial proteins, we also identified proteins inhibiting oligodendroglial differentiation such as metalloendopeptidase BMP 1 [41], thrombospondin 1 [48], and tenascin C [49]. An equilibrated action of positive and negative regulators might be necessary for a timed and successful cell fate acquisition, thereby contributing to successful establishment of new myelin sheaths. On the other hand, future studies will show whether blocking such proteins could also increase the efficiency of the MSC-CM treatment.

Finally, one of the most promising candidate proteins was TIMP-1, on the basis of its neuroprotective functions by means of matrix metalloproteinase (MMP) regulation [65] and its capacity to promote oligodendrocyte differentiation and myelination [42,43,50]. As we demonstrated that neutralization of TIMP-1 in MSC-CM results in a significantly reduced number of CNPase-positive cells and an increased (non-significant) degree of GFAP-positive cells in culture, we provide a proof-of-principle that the chosen secretome/proteome approach is valid. Moreover, we demonstrated that TIMP-1 is one of the key players in MSC-mediated oligodendrocyte differentiation and myelination.

Given the large number of differentiation-related proteins identified in the MSC secretome, we suggest that the MSC-CM effect depends on the interplay of both pro-oligodendroglial as well as anti-astrocytic factors possibly acting at different differentiation stages of which TIMP-1 constitutes one element. Upcoming experiments will therefore have to functionally investigate further protein candidates and also use combinations of neutralization antibodies or recombinant proteins for validation. Finally, given our observation that, in vivo, a much shorter instruction period by MSC-derived factors is sufficient for successful oligodendrogenesis, myelination, and cell integration, researchers should then test combinations of presumably active recombinant proteins on pre-stimulated aNSCs to be transplanted in vivo.

## 4. Materials and Methods 

### 4.1. Animals 

For the isolation of rat aNSCs and MSCs, female Wistar rats (8–10 weeks old, ≈210 g) were used throughout the study. Transplantation experiments into wild type female mice (C57Bl/6, 13–14 weeks old, ≈18–21g) and rats (Wistar, 10–12 weeks old, between 210 and 230 g) were all approved by the LANUV (Landesamt für Natur, Umwelt und Verbraucherschutz Nordrhein-Westfalen; Az.: 84-02.04.2015.A239; Az.: 84-02.04.2015.A525) and carried out in accordance with ethical care. All rodents were housed in a pathogen-free facility with 12 h light/dark cycle, and were supplied with food/water ad libitum.

### 4.2. Mesenchymal Stem Cell Culture

Preparation and cultivation of adult rat mesenchymal stem cells (MSCs) were performed as previously described [19,20]. For experiments, we cultured bone marrow-derived rat MSCs for 3 to 4 days in α-MEM/10% FBS (both Gibco Cell Culture, Life Technologies, Karlsruhe, Germany) containing 1% penicillin–streptomycin (Gibco) until reaching a confluent cell layer (90–100%). Afterwards, medium was changed and cells were cultured in α-MEM/10% FBS for another 3 to 4 days. The corresponding supernatant was filtered (0.2 µm filtropur S filter; Sarstedt AG and Co. KG, Nümbrecht, Germany) and used as rat mesenchymal stem cell-conditioned medium (MSC-CM) for aNSC stimulation. MSCs were used from passage 3 to 10.

### 4.3. Adult Rat Neural Stem Cell Culture and Transfection

Preparation and cultivation of aNSC derived from the rat subventricular zone (SVZ) were conducted as previously described [20,63]. SVZ-derived aNSCs were resuspended in neurobasal (NB) medium (Gibco) containing B27 (Gibco), 2 mM L-glutamine (Gibco), 1% penicillin–streptomycin (Gibco) supplemented with 2 mg/mL heparin (Sigma-Aldrich Chemie GmbH, Steinheim, Germany), 20 ng/mL fibroblast growth factor 2 (FGF-2; R&D Systems, Wiesbaden-Nordenstadt, Germany), and 20 ng/mL epidermal growth factor (EGF; R&D Systems, Wiesbaden-Nordenstadt, Germany), from here on referred to as NBall medium. For experiments, aNSCs were seeded with a cell number of 1 × 10^6^ cells in 1 T75 culture flask in 10 mL NBall medium and cultured for 7 days at 37 °C in a humidified incubator with 5% CO_2_. The medium was exchanged twice a week, and on day 7, we passaged aNSCs using accutase (PAA Laboratories, Pasching, Austria) for separation (10–15 min at 37 °C). For differentiation, we seeded aNSC on acid-pretreated and poly-L-ornithine/laminin (100 µg/mL and 12 µg/mL, respectively, both Sigma-Aldrich Chemie GmbH)-coated 13 mm glass cover slips (6.3 × 10^4^ cells per coverslip). Cells were cultured for 24 h in NBall medium before changing to differentiation control medium (α-MEM/10% FBS) or MSC-CM. For cell visualization of transplanted cells, we co-transfected aNSC with the UbC-StarTrack plasmids pCMV-hyPBase and UbC-EGFP [66] using the rat neural stem cell nucleofector kit (Lonza, Basel, Switzerland), as described previously [20]. Transfected cells were seeded with a concentration of 3 × 10^6^ cells in 1 T75 culture flask in 10 mL NBall medium and were cultured for 7 days at 37 °C in a humidified incubator with 5% CO_2_. Two or four days before transplantation, we seeded aNSCs on poly-L-ornithine/laminin (100 µg/mL and 12 µg/mL, Sigma-Aldrich Chemie GmbH)-coated Petri dishes (35 × 10 mm) for 24 h in NBall medium before changing to control α-MEM/ 10% FBS or MSC-CM.

### 4.4. Antibody Blocking and Immunocytochemistry

For blocking experiments, we seeded aNSCs on acid-pretreated and poly-L-ornithine/laminin-coated glass cover slips (5 × 10^4^ cells per coverslip) and cultured in NBall medium for 24 h. To neutralize metallopeptidase inhibitor 1 (TIMP-1), we incubated MSC-CM with an anti-rat TIMP-1 antibody (25 µg/mL, R&D System, AF580) for 1 h at 37 °C, prior to application to aNSCs. Isotype control IgG antibody (25 µg/mL, R&D System, AB-108-C) was used as control. In addition, we also applied both antibodies to normal control medium (α-MEM / 10% FCS) in order to exclude unspecific antibody effects. After 3 days (blocking experiments) or 1–7 days (PiggyBac-transfected aNSC differentiation) of incubation with α-MEM / 10% FCS or MSC-CM, we fixed aNSCs using 4% paraformaldehyde (PFA) and subjected them to immunocytochemical staining, as previously described [20]. After primary antibodies were blocked of unspecific binding sites and permeabilized for 45 min in 1% normal goat serum (NGS; Sigma-Aldrich Chemie GmbH) and 0.1% Triton in phosphate-buffered saline (PBS), we incubated them in 1% NGS (in PBS, 0.03% Triton) at 4 °C overnight. The following antibodies were used: rabbit anti-glial fibrillary acidic protein (GFAP; 1:4000, DAKO Agilent, Santa Clara, CA, USA; research resource identifiers (RRID): AB_10013382), mouse anti-2′,3′-cyclic-nucleotide 3′-phosphodiesterase (CNPase; 1:500, Biolegend, San Diego, CA, USA; RRID: AB_2565362), chicken anti-green fluorescent protein/citrine (GFP; 1:2000; Aves Labs, Tigard, OR, US; RRID: AB_10000240), rabbit anti-neural/glial antigen 2 (NG2; 1:100; Merck Millipore, Burlington, MA, USA; RRID: AB_11213678, 1 h RT), and rat anti-myelin basic protein (MBP; 1:250, Bio-Rad Laboratories, Inc., Hercules, CA, USA; RRID: AB_325004). Primary antibody incubation was followed by 3 washing steps with PBS and incubation with secondary (anti-mouse and anti-rabbit) antibodies conjugated with either Alexa Fluor594 or Alexa Fluor488 (1:500; Thermo Fisher Scientific, Darmstadt, Germany) in 1% NGS (in PBS, 0.03% Triton) supplemented with 4’,6-diamidin-2-phenylindol (DAPI, 0.02 µL/mL; Roche Diagnostic GmbH, Mannheim, Germany) at room temperature for 90 min. Cells were mounted with Citifluor (Cilifluor, Leicester, United Kingdom) and images were taken using a Zeiss Axioplan2 microscope (Carl Zeiss AG, Jena, Germany). For quantitative analyses, we used ImageJ Software (National Institute of Health (NIH) USA) [67,68] to count the positive cells that were normalized to the total cell number (DAPI-positive cells). For statistical analysis, we applied two-way analysis of variance (ANOVA) with Bonferroni posttest by using GraphPad Prism 5.0c software.

### 4.5. Stereotactic Cell Transplantations

Rat aNSCs were detached with accutase and kept at room temperature in their respective media. For transplantation into the mouse brain, aNSCs were centrifuged for 5 min at 140× *g*, washed once in PBS, and were finally resuspended in PBS to a concentration of 1 × 10^5^ cells/μL. The transplantation procedure was performed as previously described [26]. Briefly, recipient C57Bl/6J mice were deeply anesthetized using isoflurane or sevoflurane inhalation. The skull was exposed using a scalpel, and holes were drilled at 0.7 mm (anterior–posterior), ± 1 mm (medial–lateral). Approximately 0.75 μL of the cell suspension was injected in the white (corpus callosum) and grey (cortex) matter of the mice brains (13–14 weeks old) according to [55]. Transplantations were performed with a Hamilton syringe (10 μL Neuros Model 1701 RN, ga 33, L 0–20 mm) at 0.7 mm (anterior–posterior), ± 1 mm (medial–lateral), 2.1 to 1.8 mm (dorsal–ventral) relative to Bregma using a motorized robot stereotaxic instrument and StereoDrivesoftware (Neurostar, Tubingen, Germany). Postoperative care comprised an analgesic treatment (Rimadyl, Pfizer Deutschland GmbH, Berlin, Germany; 5 mg/kg) for 3 days, starting on the day of operation. For tissue collection, mice were deeply anesthetized with isoflurane and transcardially perfused with 20 mL cold PBS followed by 20 mL 4% PFA. Mouse brains were post-fixed overnight in 4% PFA at 4 °C, followed by 24 to 48 h cryoprotective dehydration in 30% sucrose (in PBS) at 4 °C. Brains were embedded in Tissue-Tek OCT (Sakura Finetek Europe, The Netherlands), frozen, and stored at −80 °C until preparation of 10 μm sections using a cryostat (Leica CM3050S). Sections were stored at −80 °C. 

For spinal cord transplantations, adult female rats were treated as previously described [26,69] with slight modifications. Briefly, rat aNSC transplantation was performed at thoracic level 8 (Th8) using a Hamilton syringe (10 μL Neuros Model 1701 RN, ga 33, L 0–20 mm) attached to a Small Animal Stereotaxic Instrument (David Kopf Instruments, Tujunga, CA, USA). Injections were performed at 0.1 mm lateral to the midline and 1.1 to 0.8 mm dorsal–ventral from the dural surface. At each transplantation site, we slowly and within 4 min injected 1 µL of either 1 × 10^5^ control α-MEM pre-stimulated (3 day) or MSC-CM pre-stimulated (3 days) aNSCs in PBS. Postoperative care included prophylactic oral antibiotic treatment (Baytril, Bayer Health Care, Leverkusen, Germany; 0.4 mL/kg) for 7 days and analgesic treatment (Rimadyl, Pfizer, New York, NY, USA; 5 mg/kg) for 3 days starting, on the day of surgery. After 14 or 28 days post-operation, animals were deeply anesthetized using a mixed solution containing ketamine (100 mg/kg body weight) and xylazine (10 mg/kg body weight), and were transcardially perfused with 200 mL 4 °C ice-cold PBS followed by 400 mL 4% PFA. Spinal cords were harvested and post-fixed overnight in 4% PFA at 4 °C, followed by 24 h washing in PBS and 24 to 48 h cryoprotection in 30% sucrose (in PBS) at 4 °C. Spinal cords were then embedded in Tissue-Tek OCT (Sakura Finetek Europe, Netherlands), frozen, and stored at −30 °C until preparation of 14 μm sections using a cryostat (Leica, Wetzlar, Germany, CM3050S). Sections were stored at −30 °C.

### 4.6. Immunohistochemistry

Immunohistochemical staining was performed as previously described [26]. Briefly, defrosted brain and spinal cord sections were air-dried for at least 15 min at room temperature (RT), and were afterwards rehydrated for 5 min in distilled water, transferred to −20 °C acetone (5 min), and washed in 1x tris-buffered saline (TBS) (pH 7.6) and 1x TBS-T (TBS containing 0.02% Triton) for 5 min each. For blocking, we used 10% biotin-free bovine serum albumin (BSA; Carl Roth, Karlsruhe, Germany, in TBS-T) for 30 min at RT, followed by application of the following antibodies (in 5 to 10% BSA in TBS) and incubation overnight: rabbit anti-oligodendrocyte transcription factor 2 (Olig2; in brain 1:2000, in spinal cord 1:1000; Merck Millipore, Burlington, MA, USA; RRID: AB_570666, RT), rabbit anti-neural/glial antigen 2 (NG2; 1:100; Merck Millipore, Burlington, MA, USA; RRID: AB_11213678, RT), rabbit anti-glutathione-S-transferase-π (GSTπ; 1:4500; ENZO Life Sciences, GmbH, Lörrach, Germany; RRID: AB_10615079; 4 °C), rabbit GFAP (1:10000; DAKO Agilent, Santa Clara, CA, USA; RRID: AB_10013382; 4 °C), rat anti-MBP (1:500, Bio-Rad Laboratories, Inc., Hercules, CA, USA; RRID: AB_325004, RT or 4 °C), chicken anti-green fluorescent protein/citrine (GFP; 1:2000; Aves Labs, Tigard, OR, USA; RRID: AB_10000240; 4 °C or RT), mouse anti-Caspr (anti-Caspr/paranodin/neurexin IV, clone K65/35; 1:500; 75-001; UC Davis/NIH NeuroMab Facility, CA, USA; RRID: AB_10671175; 4 °C), rabbit anti-neurofilament (NF; 1:2500; Abcam, Cambridge, United Kingdom; RRID: AB_306298; 4°C), and rabbit anti-neuronal nuclei antigen (NeuN; 1:1000; Abcam, Cambridge, United Kingdom; RRID: AB_2532109; 4 °C).

Sections were washed 2 times in TBS for 5 min and incubated with appropriate secondary antibodies (goat anti-chicken; goat anti-rabbit; goat anti-rat conjugated with either Alexa Fluor 594, 488, 405, or 647; 1:200 in TBS; Thermo Fisher Scientific, Darmstadt, Germany) for 30 min at RT. DAPI (0.04 µl/mL; Roche Diagnostic GmbH) or RedDot 2 (1:200; Biotium, Fremont, CA, USA; cat. #: 40061) were included for nuclei labelling. Slices were mounted with Immu-Mount (Thermo Fisher Scientific, Darmstadt, Germany) and analyzed using a confocal confocal laser scanning microscopy (CLSM) microscope 510 (CLSM 510, Carl Zeiss AG, Oberkochen, Germany). Immunohistochemical staining on brain and spinal cord sections was performed using the corresponding centers of transplantation. An average of 25 brain sections and 13 spinal cord sections per marker and per time-point were analyzed, including 3 brain slices or 2 spinal cord slices per each slide. Fluorescently labeled cells were counted on each picture/section, and the mean number per slide (containing 3 brain or 2 spinal cord sections, respectively) was calculated, leading to an average value for each animal.

### 4.7. Immunoelectron Microscopy

Immunoelectron microscopy was performed as previously described [26], with slight modifications. Briefly, adult rats were perfused with 4% PFA in 1 M cacodylate buffer (Carl Roth GmbH + Co. KG, Karlsruhe, Germany), and spinal cords were harvested and post-fixed overnight in 4% PFA in 1M cacodylate buffer. After post-fixation, spinal cords were washed over night in PBS and embedded in 6% agarose in cacodylate buffer. Coronal sections (50 µm) were cut in PBS using a microtome (Microm HM 650 V, Thermo Fisher Scientific, Waltham, MA, USA). Free-floating sections were blocked with 1% BSA in PBS and incubated with 0.1 M NaIO_3_ in PBS and subsequently in 5% dimethyl sulfoxide in PBS. Sections were incubated with rabbit anti-GFP antibody (1:100; AB3080, Merck Millipore, Burlington, MA, USA; RRID: AB_91337) in 1% BSA in PBS overnight at 4 °C, and immune reactions were subsequently visualized using a biotinylated secondary antibody (biotinylated goat anti-rabbit IgG; 1:50; BA-1000, Vector Laboratories, Burlingame, CA, USA; RRID: AB_2313606) and streptavidin–biotin–peroxidase (PK-6100, Vector Laboratories, Burlingame, CA, USA; RRID: AB_2336819) complex using diaminobenzidine–HCl (SK-4105, Vector Laboratories, Burlingame, CA, USA; RRID: AB_2336520) and H_2_O_2_. After diaminobenzidine staining, appropriate transplantation regions were cut, and sections were osmicated and processed for light and electron microscopy by dehydration and embedding in Spurr’s medium. Ultrathin sections (70 nm) were mounted to copper grids, counterstained with lead citrate, and investigated using a ProScan Slow Scan CCD camera connected to a Leo 906 E electron microscope (Zeiss, Jena, Germany) and corresponding software iTEM (Soft Imaging System). Transplanted cells were identified on the basis of GFP immunoreactivity (indicated by dark precipitates).

### 4.8. Statistical Analysis of IHC and ICC

Statistical analyses and graphs were generated using Excel and Graph-Pad Prism 5.0 software. To determine statistical significance in graphs with more than two conditions, we applied ordinary two-way analysis of variance (ANOVA) with Bonferroni posttest. Statistical significance thresholds were set as follows: * *p* < 0.05; ** *p* < 0.01; *** *p* < 0.001. All data are shown as mean values ± SEM, and “*n*” represents the number of independent experiments performed.

### 4.9. Preparation of Mesenchymal Stem Cell Secretome and Proteome

Mesenchymal stem cells were prepared, as described previously, through seeding 4 × 10^5^ cell/100 mm Ø culture dish in α-MEM-10% FBS until a confluent cell layer was achieved. For mass spectrometry analysis, we used four independent biological replicates of MSCs. Confluent MSCs were washed three times with PBS in order to remove the FBS and were then incubated for another 48h in serum-free N2-medium [22,70,71] consisting of Dulbecco’s modified Eagle’s medium (DMEM, Gibco Cell Culture, Thermo Fisher Scientific, Darmstadt, Germany) containing 5 mg/mL insulin, 30 nM sodium selenite, 100 µM putrescine, 20 nM progesterone, and 5 µg/mL transferrin (all Sigma-Aldrich Chemie GmbH). Medium was changed again after 48 h to further exclude FBS effects, and was then incubated for another 48 h in N2 medium.

To analyze secreted proteins within this second N2 medium preparation by mass spectrometry, we performed trichloroacetic acid (TCA) precipitation as previously described [70,71]. Briefly, conditioned medium was transferred to a 50 mL tube, centrifuged at 950× *g* at 4 °C for 10 min, and filtered through a 0.2 µm filter (Pall Acrodisc). The medium was mixed with 10% sodium lauroyl sarcosinate (Sigma-Aldrich Chemie GmbH) up to a final concentration of 0.1%. Ice-cold trichloroacetic acid (Sigma-Aldrich Chemie GmbH) was added (7.5% final concentration). The medium was incubated for 1 h on ice and then centrifuged at 7100× *g* at 4 °C for 10 min. After removing the supernatant, we resuspended the pellet in 1 mL ice-cold acetone and we then centrifuged it again. After removing the supernatant, we dried the pellet at room temperature and then dissolved it in lysis buffer consisting of 30 mM Tris base (Sigma-Aldrich Chemie GmbH), 7M urea (Sigma-Aldrich Chemie GmbH,), and 2 M thiourea (Sigma-Aldrich Chemie GmbH). The protein content was determined by Pierce 660 nm Protein Assay (Thermo Fisher Scientific).

To analyze the corresponding cell proteome, we washed MSCs 3 times with cold PBS after removing the conditioned medium for secretome analysis. Afterwards, 2 mL cold PBS was added and cells were detached using a cell scraper. Cell suspension was transferred to a 15 mL tube and centrifuged with 800× *g* at 4 °C for 5 min. The supernatant was removed, and cell pellets were immediately frozen and stored at −80 °C. MSCs were lysed by using the lysis buffer described above. The lysate was sonicated 6 × 10 s, centrifuged at 16,000× *g* for 15 min, and the protein concentration of the supernatant was then determined.

For in-gel digestion, 5 µg total protein of each sample (secretomes as well as proteomes) was used for short SDS gel electrophoresis (5 mm running distance, 10 min) and stained with Coomassie brilliant blue. The resulting lanes were excised, washed, reduced with 10 mM dithiothreitol (DTT; Serva Electrophoresis, Heidelberg, Germany) and alkylated with 55 mM iodacetamide (Sigma-Aldrich Chemie GmbH). Proteins were digested with 0.1 µg trypsin (Serva Electrophoresis) overnight at 37 °C, and peptides were extracted, dried, and finally resuspended in 0.1% trifluoroacetic acid.

### 4.10. Liquid Chromatography and Mass Spectrometric Analysis

Extracted peptides were separated by an Ultimate 3000 RSCLnano System (Thermo Fisher Scientific) with an Acclaim PepMap100 trap column (3 µm C18 particle size, 100 Å pore size, 75 µm inner diameter, 2 cm length, Thermo Fisher Scientific) as a precolumn using 0.1% trifluoroacetic acid (TFA) as a mobile phase and an Acclaim PepMapRSLC (2 µm C18 particle size, 100 Å pore size, 75 µm inner diameter, 25 cm length, Thermo Fisher Scientific) analytical column. The flow rate for analytical separation was constant with 300 nL/min, and a 2 h gradient of 0.1% formic acid (Fluka) to 0.1% formic acid/60% acetonitrile was used for peptide separation. Peptides were eluted via nano electrospray ionization into the mass spectrometer (Orbitrap Fusion Lumos mass spectrometer, Thermo Fisher Scientific) operated in positive mode with advanced peak determination enabled. Precursor mass spectra were recorded in the orbitrap analyzer within a mass range of 200–2000 *m*/*z* and a resolution of 120,000 (maximum ion time: 60 ms, automatic gain control target value: 250,000, profile mode). For a maximum of 2 s, we isolated precursors with charge states +2 to +7 and a minimum intensity of 5000 within a 1.6 *m*/*z* isolation window and fragmented via higher-energy collisional dissociation. MS/MS spectra were recorded in the linear ion trap in centroid mode with a maximal ion time of 50 ms and a target value for the automatic gain control was set to 10,000. The scan rate was “rapid”, and already fragmented precursors were excluded from further isolation for the next 60 s.

### 4.11. Analysis of Mass Spectrometric Data

To identify peptides and proteins, we used the MaxQuant environment (version 1.6.6.0, MPI for Biochemistry, Planegg, Germany). If not stated otherwise, the identification was done with standard parameters. Searches were performed using the rat UP000002494 proteome dataset (29,951 entries) downloaded on 10th April 2019 from the UniProt Knowledgebase using tryptic specificity (cleavage behind R and K) with a maximum of 2 missed cleavages sites. Carbamidomethylation at cysteines was considered as fixed, and methionine oxidation was set as variable modification. A first search was performed with 20 ppm precursor mass tolerance. Peptides identified with high confidence were then used for recalibration using the “software lock mass” feature of MaxQuant [72]. Thereafter, a main search was conducted with a precursor mass tolerance of 4.5 ppm. The mass tolerance or fragment spectrum was set to 0.5 Da. Peptides and proteins were accepted at a false discovery rate of 1%. The “match between runs” function was enabled as well as label-free quantification.

Corresponding secretomes and proteomes were compared to identify enriched proteins in the secretome, probably representing secreted proteins according to Grube et al. [29]. After a MaxQuant-based database search and quantification, we analyzed quantitative protein level data within the Perseus framework (version 1.6.6.0, MPI for Biochemistry, Planegg, Germany). Proteins identified only by site or marked as contaminant (from the MaxQuant contaminant list) as well as reverse hits were excluded. Proteins with at least 2 identified peptides and a minimum of 3 valid values in at least 1 group were considered. Calculations were done on normalized intensities (LFQ intensities), as provided by MaxQuant, and missing data were imputed before statistical analysis by values from a downshifted normal distribution (width 0.3 standard deviations, down-shift 1.8 standard deviations). The significance analysis of microarrays (SAM) method [73] was applied on log_2_-transformed values using a S0 constant of 0.8 and a 5% false discovery rate-based cutoff. Presented fold changes were calculated as difference from mean values of log_2_-transformed intensities.

To predict unconventionally secreted proteins, we used the OutCyte prediction tool [74], wherein we selected classically secreted proteins on the basis of the annotation from UniProtKB (signal peptide predicted, but neither a transmembrane domain nor a kdel sequence present). Gene Ontology (GO) biological process (GOBP) and cellular compartment (GOCC) were used for categorical annotations of proteins identified in the MSC-derived secretome, and annotation enrichments were calculated by Fisher’s exact tests. Enrichment associated *p*-values were adjusted via the method of Benjamini and Hochberg.

### 4.12. Data Availability

The mass spectrometry proteomics data were deposited to the ProteomeXchange Consortium via the PRIDE [75] partner repository with the dataset identifier PXD018231. 

## 5. Conclusions

In the present study, we demonstrated that MSC-derived secreted factors promote oligodendrogenesis of aNSCs transplanted into different CNS regions. Furthermore, mass spectrometry-based secretome analysis provided insights into the underlying mechanisms. Our analysis identified several proteins previously shown to regulate oligodendrogenesis. As a promising protein candidate, we validated TIMP-1 to be an active component of the MSC-conditioned medium promoting oligodendrogenesis in vitro. Given that MSC-derived factors are sufficient to promote successful oligodendrogenesis, myelination and cell integration, transplantation of pre-stimulated aNSCs upon CNS injury, and demyelination could help to promote functional remyelination.

## Figures and Tables

**Table 1:** Mesenchymal stem cell-secreted proteins associated with oligodendroglial lineage regulation.

ID	Oligodendroglial Lineage-Promoting Factor	Gene Name	Description	Secretion Via/Localization	Reference
P07897	Aggrecan core protein	Acn	Inhibits oligodendrocyte myelination	SP ^1^	[30]
A0A140TA94	Chordin	Chrd	Promotes oligodendrogenesis From subventricular zone derived stem cells in vitro and in vivo	SP	[31]
Q9R1E9	Connective tissue growth factor	Ctgf	Negatively regulates myelination	SP	[32]
F1M8K0	Dystroglycan 1	Dag1	Intracellular portion of cleaved dag promotes oligodendroglial precursor cell proliferation/regulates myelin membrane production, growth, or stability	SP	[33,34]
A0A096P6L8	Fibronectin	Fn	Maintenance and proliferation of OPCs	SP	[35]
Q63772	Growth arrest-specific protein 6	Gas6	Protects oligodendrocytes from tumor necrosis factor-α-induced apoptosis	SP	[36]
P12843	Insulin-like growth factor-binding protein 2	Igfbp2	Negative effector of oligodendrocyte survival and differentiation	SP	[37]
F1M9B2	Insulin-like growth factor-binding protein 7	Igfbp7	Inhibits the differentiation of oligodendrocyte precursor cells via regulation of wingless-related integration site (Wnt) / β-catenin signaling	SP	[38]
P12843	Laminin subunit beta-2	Lamb2	Extracellular matrix proteins known to be important for myelination	SP	[39,40]
F1M798	Metalloendopeptidase	BMP1	Inhibits oligodendrogenesis	SP	[41]
P30120	Tissue inhibitor of metalloproteinase type 1	Timp-1	Promotes oligodendrocyte differentiation and central nervous system myelination	SP	[42,43]
P08721	Osteopontin	Spp1	Stimulates MBP synthesis and myelin sheath formation in vitro	SP	[44]
F7EPE0	Sulfated glycoprotein 1/prosaposin	Psap	Known as a myelinotrophic factor protecting myelinating glial cells	SP	[45,46]
Q9JI92	Syntenin-1	Sdcbp	Plays a role in OPC migration	UPS ^2^	[47]
M0R979	Thrombospondin 1	Thbs1	Reduces oligodendrogenesis	SP	[48]
A0A0G2K1L0	Tenascin c	Tnc	Inhibits OPC differentiation and myelination	SP	[49]

^1^ SP: signal peptide; ^2^ UPS: unconventional protein secretion.

## Supplementary Materials

Supplementary materials can be found at https://www.mdpi.com/1422-0067/21/12/4350/s1.

## Abbreviations

α-MEM	Alpha-minimum essential medium
Acan	Aggrecan
ANOVA	Analysis of variance
aNSCs	Adult neural stem cells
BMP	Bone morphogenetic protein
BSA	Bovine serum albumin
Caspr	Contactin-associated protein
Chrd	Chordin
CLSM	Confocal laser scanning microscopy
CNPase	2′,3′-Cyclic-nucleotide 3′-phosphodiesterase
CNS	Central nervous system
CTGF	Connective tissue growth factor
Dag1	Dystroglycan
DAPI	4´,6-diamidin-2-phenylindol
DMEM	Dulbecco’s modified Eagle’s medium
DTT	Dithiothreitol
ECM	Extracellular matrix
EF	Enrichment factor
EGF	Epidermal growth factor
EM	Electron microscopy
FCS	Fetal calf serum
FDR	False discovery rate
FGF	Fibroblast growth factor
Fn	Fibronectin
Gas6	Growth arrest-specific protein 6
GFAP	Glial fibrillary acidic protein
GFP	Green fluorescent protein
GM	Grey matter
GSTπ	Glutathione-S-transferase-π
GO	Gene Ontology
GOBP	Gene Ontology biological process
GOCC	Gene Ontology cellular component
IGFBP	Insulin-like growth factor-binding protein
IgG	Immunoglobulin G
Lamb2	Laminin subunit beta-2
LC–MS/MS	Liquid chromatography–mass spectrometry/mass spectrometry
MBP	Myelin basic protein
MMP	Matrix metalloproteinase
MSCs	Mesenchymal stem cells
MSC-CM	Mesenchymal stem cell conditioned medium
NB	Neurobasal
NF	Neurofilament
NG2	Neural/glial antigen 2
NGS	Normal goat serum
Olig2	Oligodendrocyte transcription factor 2
OPCs	Oligodendrocyte progenitor cells
PBS	Phosphate-buffered saline
PFA	Paraformaldehyde
ps	Pre-stimulated
Psap	Prospaposin
pt	Post transplantation
Sdcbp	Syntenin-1
SP	Signal peptide
Spp1	Osteopontin
SVZ	Subventricular zone
TCA	Trichloroacetic acid
Thbs1	Thrombospondin 1
TIMP-1	Tissue inhibitor of metalloproteinase type 1
TM	Transmembrane
Tnc	Tenascin c
UPS	Unconventional protein secretion
WM	White matter
