# Supplementary material for: Secretome Analysis of Mesenchymal Stem Cell Factors Fostering Oligodendroglial Differentiation of Neural Stem Cells In Vivo
Source: Int J Mol Sci. 2020 Jun 18;21(12):4350. doi: 10.3390/ijms21124350 (PMC7352621; doi:10.3390/ijms21124350)
Supplement: Supplementary file 1 [file ijms-21-04350-s001.zip › ijms-829876-supplementary final.pdf]

Secretome analysis of mesenchymal stem cell factors fostering oligodendroglial differentiation of neural stem cells in vivo

Iria Samper Agrelo, Jessica Schira-Heinen, Felix Beyer, Janos Groh, Christine Bütermann, Veronica Estrada, Gereon Poschmann, Ana Bribian, Janusz J. Jadasz, Laura Lopez-Mascaraque, David Kremer, Rudolf Martini, Hans Werner Müller, Hans Peter Hartung, James Adjaye, Kai Stühler and Patrick Küry

Supplementary Figures S1-S4

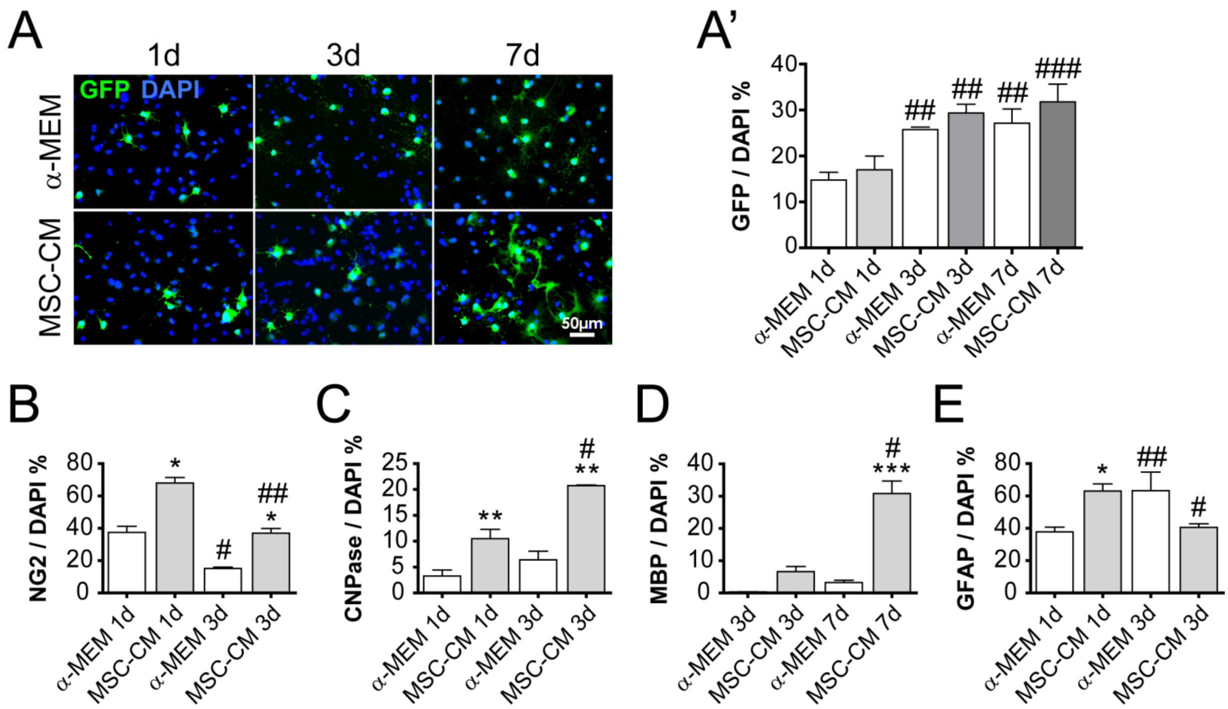

Figure S1. Distribution of GFP-PiggyBac transfected cells after MSC-CM stimulation in culture. (A) Immunocytochemical staining of GFP-PiggyBac transfected aNSCs stimulated with α-MEM or MSC-CM (A') showed the same degree and the same increase over the time of GFP-positive aNSCs under both conditions. NG2- (B), CNPase- (C), MBP- (D) and GFAP-positive (E) cells at 1 and 3 or 7 days after pre-stimulation relative to DAPI-positive nuclei showing the same distribution as previously described for non-transfected aNSCs [20]. Data are shown as mean values ± SEM derived from n=3 experiments. For statistical analysis, a two-way ANOVA with Bonferroni posttest was used with a significant threshold set as *p ≤ 0.05, ** p ≤ 0.01, ***p ≤ 0.001 for comparison of control to MSC-CM treated cells; # p ≤ 0.05, ## p ≤ 0.01, ###p ≤ 0.001 for comparison of the analyzed time points.

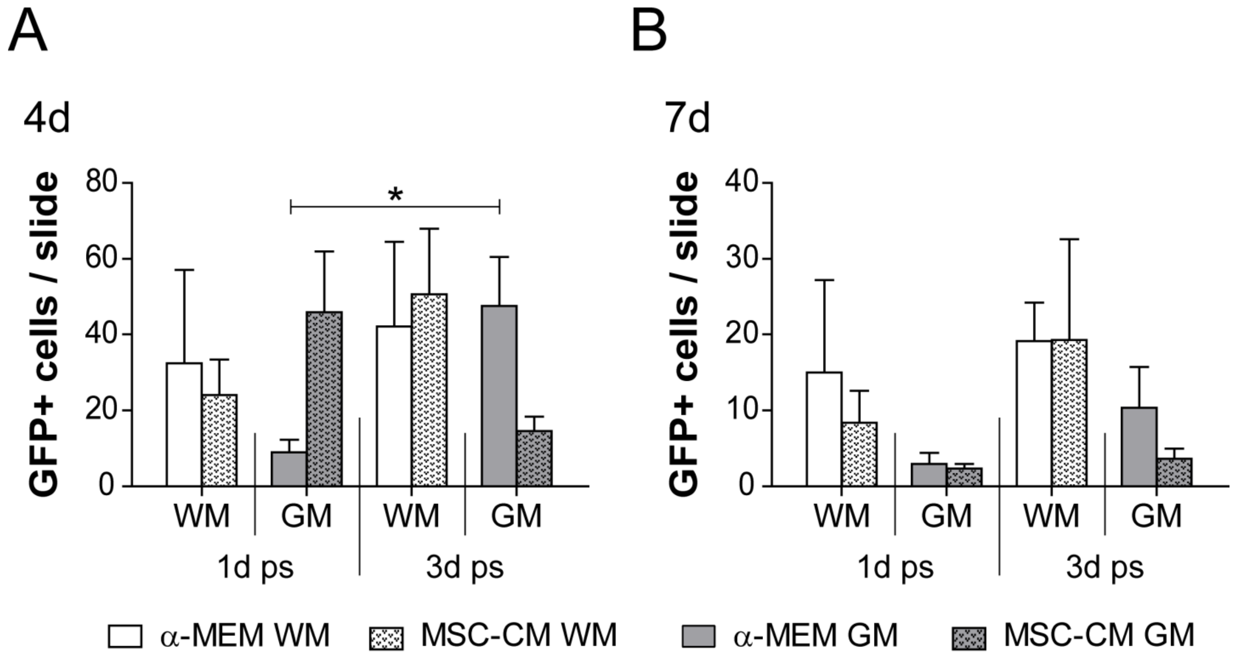

Figure S2. Survival rates of GFP-expressing cells after transplantation into the grey and white matter of the mouse brain. In general, no significant difference in the number of GFP-positive cells per slide between grey and white matter was detected after 4 days pt (A) and 7d pt (B) under both conditions and following both pre-stimulation periods. Comparison of 1d to 3d pre-stimulation revealed that α-MEM treated cells survive better within the grey matter. Statistical significance was calculated using a two-way ANOVA with Bonferroni posttest with a threshold set at * $p \leq 0.05$, compared 1d ps to 3d ps. Animal number (n): 3-5.

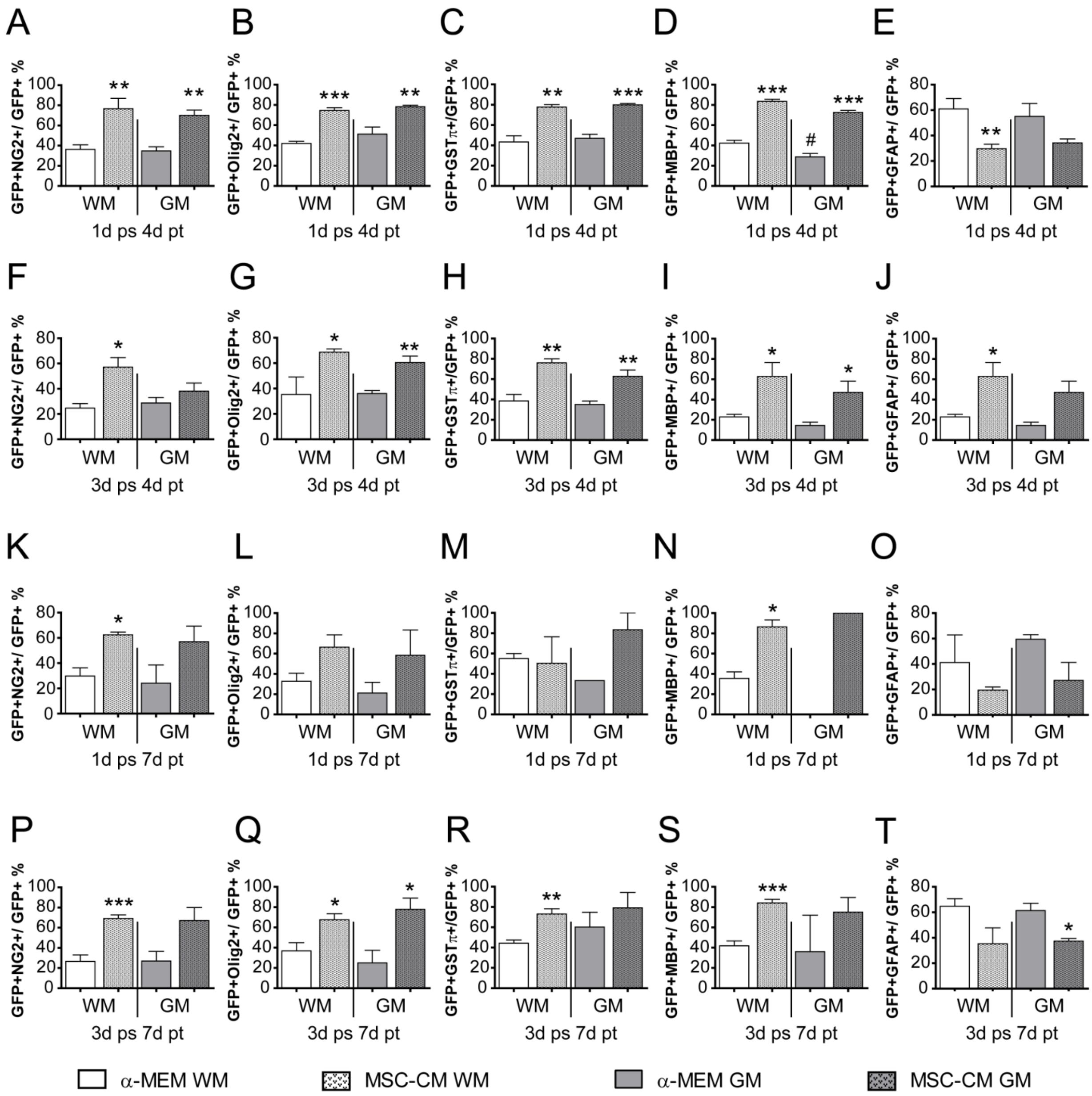

Figure S3. MSC-CM pre-stimulated aNSCs differentiate into mature oligodendrocytes independent of their transplantation location. (A-J) Quantitative analysis of GFP-positive cells transplanted either into the grey or white matter of the mouse brain showed no differentiation difference between these two regions after 4 days post transplantation (pt). (K-T) This was also evident for transplanted cells after 7 days pt. For statistical analysis, a two-way ANOVA with Bonferroni posttest was used: * $p \leq 0.05$, ** $p \leq 0.01$, *** $p \leq 0.001$, MSC-CM compared to the respective α-MEM control (1 or 3 day pre-stimulation); # $p \leq 0.05$, compared white to grey matter (α-MEM or MSC-CM). Animal numbers (n): 3-5.

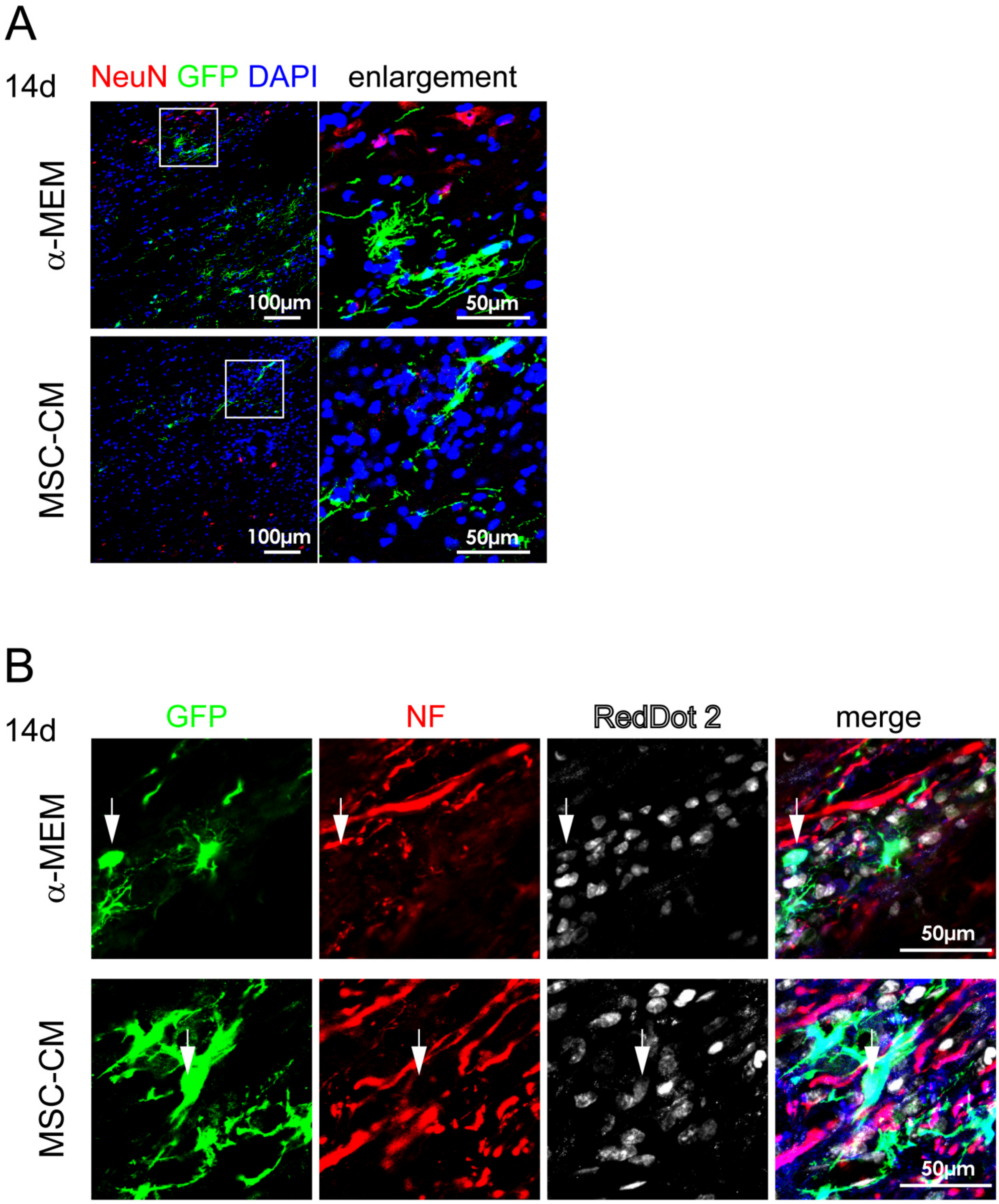

Figure S4. Pre-stimulated aNSCs do not differentiate into the neuronal lineage after transplantation into the spinal cord. (A) Representative images of GFP-positive cells (green) revealed no co-localization with NeuN (red) at 14d pt independent of the pre-treatment. (B) Immunohistochemical staining for neurofilament (NF) also revealed no GFP/NF-double positive cells after transplantation into the rat spinal cord. Arrows point to GFP-positive (green) and marker-negative cells. Animal numbers (n): 4-5.
