# Supplementary material for: Secretome Analysis of Mesenchymal Stem Cell Factors Fostering Oligodendroglial Differentiation of Neural Stem Cells In Vivo
Source: Int J Mol Sci. 2020 Jun 18;21(12):4350. doi: 10.3390/ijms21124350 (PMC7352621; doi:10.3390/ijms21124350)
Supplement: Supplementary file 1 [file ijms-21-04350-s001.zip › Table S1 description.docx]

**Table S1**: Quantitative secretome and proteome data including *bona fide* secreted proteins (152, yellow), protein identification data (rows D-L), protein annotations from UniProt KB and OutCyte predictions (rows M-V), statistical analysis for proteome/secretome comparison (rows W-AC) and quantification data including mean protein intensities and standard deviations (rows AD-AG), number of valid values (rows AH-AI), peptide spectrum matches (rows AJ-AR) and intensities (rows AS-BA) and normalized intensities (rows BB-BI). SP = signal peptide, TM = transmembrane, UPS = unconventional protein secretion, S = secretome, P = proteome.
